# Spatial distribution of electrons near the Fermi level in the metallic LaB_6_ through accurate X-ray charge density study

**DOI:** 10.1038/srep41375

**Published:** 2017-01-25

**Authors:** Hidetaka Kasai, Eiji Nishibori

**Affiliations:** 1 Division of Physics, Faculty of Pure and Applied Sciences, Center for Integrated Research in Fundamental Science and Engineering & Tsukuba Research Center for Interdisciplinary Materials Science, University of Tsukuba. 1-1-1, Tennodai, Tsukuba, Ibaraki, 305-8571, Japan

## Abstract

Charge densities of iso-structural metal hexaborides, a transparent metal LaB_6_ and a semiconductor BaB_6_, have been determined using the *d* > 0.22 Å ultra-high resolution synchrotron radiation X-ray diffraction data by a multipole refinement and a maximum entropy method (MEM). The quality of the experimental charge densities was evaluated by comparison with theoretical charge densities. The strong inter-octahedral and relatively weak intra-octahedral boron-boron bonds were observed in the charge densities. A difference of valence charge densities between LaB_6_ and BaB_6_ was calculated to reveal a small difference between isostructural metal and semiconductor. The weak electron lobes distributed around the inter B_6_ octahedral bond were observed in the difference density. We found the electron lobes are the conductive *π*-electrons in LaB_6_ from the comparison with the theoretical valence charge density. We successfully observed a spatial distribution of electrons near the Fermi level from the X-ray charge density study of the series of iso-structural solids.

Solid state crystalline materials can be classified by their electrical properties, which are insulator, semiconductor, semimetal, and metal. The properties are governed by a behavior of a few electrons per unit cell in the valence band. Exotic physical properties including high temperature superconductor^1^ have often been observed in a narrow region in between two different properties such as insulator and metal. Several exotic properties have never been accompanied with the structural changes. An isostructural series of materials with different electrical properties is one of ideal system to investigate a structure-property relationship and to discover novel properties.

Metal hexaborides *M*B_6_, where *M* is alkaline earth or rare earth metal, exhibit metallic and semiconductor properties by changing the *M* ion. The Bardeen–Cooper–Schrieffer (BCS) type superconductivities of *M*B_6_ were found in LaB_6_ and YB_6_^2^. The metal hexaborides have a B_6_ octahedron in the structure. The *M* ion is located at the body-center surrounded by the B_6_ octahedra. The B_6_ octahedron has 18 valence electrons. Two electrons per B_6_ octahedron are required to fulfill the bonding orbital of B_6_. Metal hexaborides with divalent metal ions are considered to be semiconductor and with the trivalent ions are metal from the consideration^3^. Theoretical study suggests that the *π*-electron like an anti-bonding orbital of B_6_ contributes electrical conductivity in the metallic trivalent *M*B_6_^4^.

LaB_6_ is widely used as a cathode material due to the electron-emitting properties, the work function^5^, and the long lifetime. LaB_6_ also has attracted attention as a transparent conductive material^6^. These properties are closely connected to the electronic structure. The electronic structure and the bonding properties in LaB_6_ have been investigated by the advanced experimental methods such as the de Haas-van Alphen effect^7^, two dimensional angular correlation of the electron positron annihilation radiation^8^ and the electric-field gradient from the^11^B nuclear-magnetic resonance^5^. These results are consistent with theoretical band structure calculated by many researchers in different kinds of methods^9^,^10^,^11^,^12^. All the studies provide almost consistent features near the Fermi level. Hossain *et al*. reported co-existence of ionic, covalent and metallic bonds in the electronic structure of LaB_6_ system by the pseudo-potential band calculation^9^. They proposed that the co-existence can account for the high efficiency of the electron emission.

Barium atom located adjacent to La in a periodic table also forms a metal hexaboride BaB_6_. Alkaline-earth hexaboride has been considered as a simple semiconductor with band gap of several tens eV^13^. A band structure of BaB_6_ has been investigated by a linear muffin-tin orbital atomic-sphere approximation tight-binding (TB-LMTO-ASA) formalism^14^. The electronic structure of BaB_6_ has also been calculated by a generalized gradient-corrected density functional theory using ultrasoft pseudopotentials and a plane-wave basis^14^. It was found that the band gap at X point linearly depends on the atomic coordinates of boron. They suggested that the parameters easily change the electronic properties from metallic to insulating.

BaB_6_ can be a good comparative material to investigate a spatial distribution of electrons near the Fermi level in metallic hexaboride, LaB_6_. Since, BaB_6_ is isostructural with LaB_6_ and the difference of total number of electron per unit cell is only one. The valence band for LaB_6_ has large dispersion with bottom at X point in the reported band structures^9^,^10^,^11^,^12^. The smallest band gap of BaB_6_ has been found at X point from the several theoretical calculations^13^,^14^,^15^. Comparison of the electron distribution between LaB_6_ and BaB_6_, in principle, should enable us to visualize the spatial distribution of the conductive electrons in LaB_6_ from the similarities of their band structures.

The charge density distribution of a material is now one of the most accurate observable of experimental investigation in science. We have developed the high quality measurement method of X-ray powder diffraction data at SPring-8 powder diffraction beamline^16^. We recently achieved to measure diffraction data available to *d*-spacing range *d* > 0.29 Å for charge density study of LiCoO_2_^17^. The diffraction data were successfully analyzed by a maximum entropy method (MEM)^18^ and a multipole refinement^19^. The experimental procedure we developed has been widely applied to accurate charge density studies of materials such as α-boron^20^, TiO_2_^21^, Al_2_O_3_^21^, CoSb_3_^22^,^23^, silicon^24^ and diamond^24^,^25^. The technique successfully determined the detailed charge density features which were quantitatively comparable to those determined theoretically^17^,^20^,^21^,^22^,^23^,^24^,^25^. Recently, the charge density studies from ultra-high resolution synchrotron radiation powder diffractions have been reported using an all-in-vacuum diffractometer installed at PETRA-III^26^. Core electron deformations of diamond^26^, silicon^27^ and cubic boron nitride^28^, and anharmonic thermal vibration of copper^29^ have been observed from powder diffraction data. Several analytical procedures such as Wilson plot^26^,^27^,^28^,^29^, and uncertainty estimation using particle statics^27^ were used in the studies. These techniques are under development since it still depends on materials^26^,^27^,^28^.

In this study, we investigated the charge densities of divalent and trivalent metal hexaborides, semiconducting BaB_6_ and metallic LaB_6_, through the ultra-high resolution powder diffraction data. Accurate charge densities were determined by the experimental and analytical procedure using the multiple powder diffraction datasets at SPring-8 reported by Nishibori *et al*.^24^ and Svendsen *et al*.^25^ We achieved the analysis of the data with the highest reciprocal space resolution, *d* > 0.22 Å. A use of relativistic atomic scattering factors was required for analysis of *d* < 0.25 Å data.

## Results

Rietveld refinements of LaB_6_ and BaB_6_ using two datasets, D1 and D2 data, were carried out using program synchrotron powder (SP)^24^. Weak peaks from Al_2_O_3_ found in the diffraction data of BaB_6_ were treated as a second phase in the Rietveld refinement. The reciprocal resolution used in the analysis for LaB_6_ and BaB_6_ are corresponding to *d* > 0.203 Å and *d* > 0.225 Å, respectively. Reliability factors based on weighted profile, *R*_wp_, and Bragg intensity, *R*_I_, of LaB_6_ were 2.27% and 0.97% for two datasets and those of BaB_6_ were 2.22% and 1.22% for two datasets.

We extracted observed structure factors from the results of Rietveld refinements. The structure factors of completely overlapped reflections were estimated using the calculated structure factors based on the independent atom model in the Rietveld refinement. We performed multipole refinements of the observed structure factors using the program XD^30^. The calculated structure factors were updated by the multipole refinement. The ratios between structure factors of the completely overlapped reflections were changed by the multipole model. We improved extraction of the observed structure factors by a powder diffraction pattern fitting using the structure factors from the multipole refinement. The function was added to the originally developed program SP^24^. The iterative procedure of the multipole refinement and powder pattern fitting was conducted twice for LaB_6_ and three times for BaB_6_ until the changes of parameters for multipole refinement were converged within standard uncertainty.

**Figure 1:**
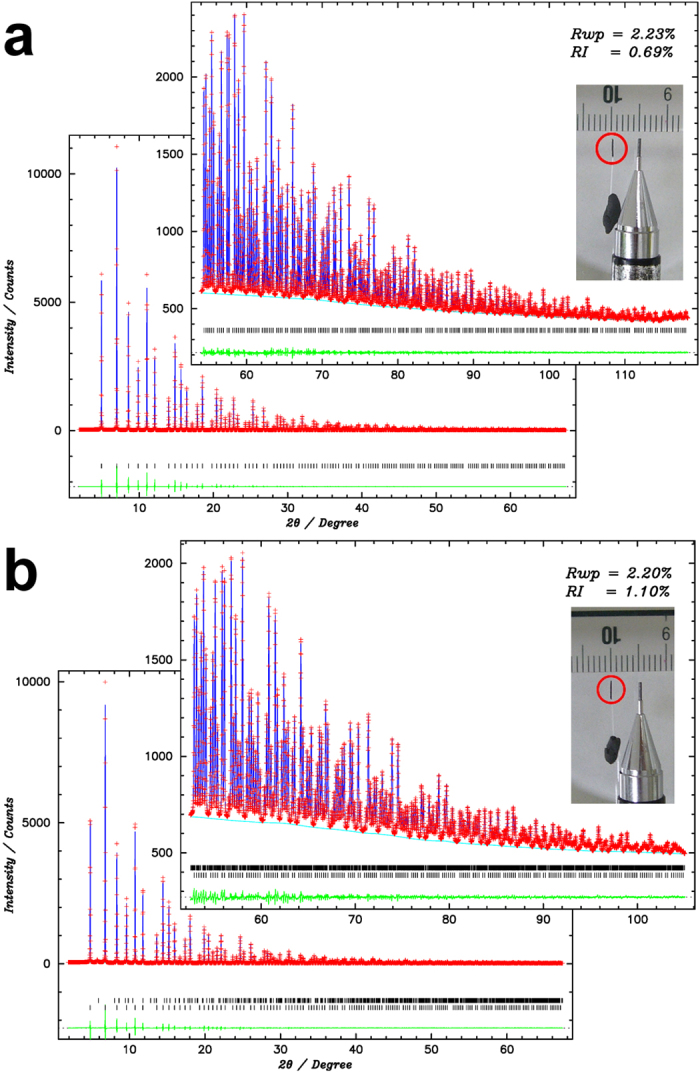
Fitting results of Rietveld refinement. (**a**) The results of LaB_6_. (**b**) BaB_6_. Insets show the results of high angle D2 data. The photographs of sample are also displayed with scaler. The graphs are drawn by the program using pgplot library (http://www.astro.caltech.edu/~tjp/pgplot/) and edited with Gimp 2.8.14 (https://www.gimp.org).

The results of the final powder fittings for LaB_6_ and BaB_6_ are shown in Fig. 1a and b, respectively. The *R*_wp_ and *R*_I_ of the final powder fittings for LaB_6_ were 6.39% and 0.56% for D1, 0.71% and 0.76% for D2, and 2.23% and 0.69% for two datasets and those of BaB_6_ were 5.92% and 0.92% for D1, 0.86% and 1.20% for D2, and 2.20% and 1.10% for two datasets. The structure factors extracted based on the fittings were used for the charge density studies by the multipole refinement and MEM.

We detected anharmonic thermal vibrations of boron atoms from a residual density of LaB_6_ during process of the refinement. The parameters of the anharmonic thermal vibrations of boron atoms for LaB_6_ were successfully determined in the multipole refinement. The reliability factors, *R* and *R*_W_, on the final multipole refinement were as small as 0.47% and 0.40% for LaB_6_ and 0.57% and 0.42% for BaB_6_. Those values are much smaller than those of the Rietveld refinement.

Table 1 shows the refined multipole populations and κ parameters for LaB_6_ and BaB_6_. The anharmonic thermal vibrations by Gram-Charlier temperature factor formalism of B for LaB_6_ are also listed in Table 1. The residual density maps of harmonic and anharmonic thermal parameter model are shown in Supplementary Figure S1. The decrease of the residual in antibond direction of B atom is recognized in the maps. Several parameters relating to the heavy elements, La and Ba, were individually refined and fixed due to their unstable behavior in the refinement. The *P*_v_ parameters of Ba and B in BaB_6_ were individually refined, then the other multipole parameters were refined. The κ and κ’ of La were fixed to 1.0 since the parameters became physically meaningless negative values by the refinement. The result for BaB_6_ shows populations of *P*_20_ and *P*_30_ mainly contribute to chemical bonding between boron atoms. The other populations are less than 20% of *P*_20_ and *P*_30_ in BaB_6_. Similar large *P*_20_ and *P*_30_ populations are found in LaB_6_. The populations of *P*_10_, *P*_40_, and *P*_44+_ for LaB_6_ are at least twice larger than those of BaB_6_.

We investigated charge densities of LaB_6_ and BaB_6_ by the multipole refinement, the MEM analysis, and the theoretical calculations in the present study. In comparison of LaB_6_ and BaB_6_, there was no significant difference in charge densities between *M* and B. The charge densities between *M* and B are shown in the Supplementary Figures S2, S3, and S4. The significant difference between BaB_6_ and LaB_6_ was found in the bonding network between boron atoms. The bonding nature of boron atoms is mainly discussed in the following text.

**Figure 2:**
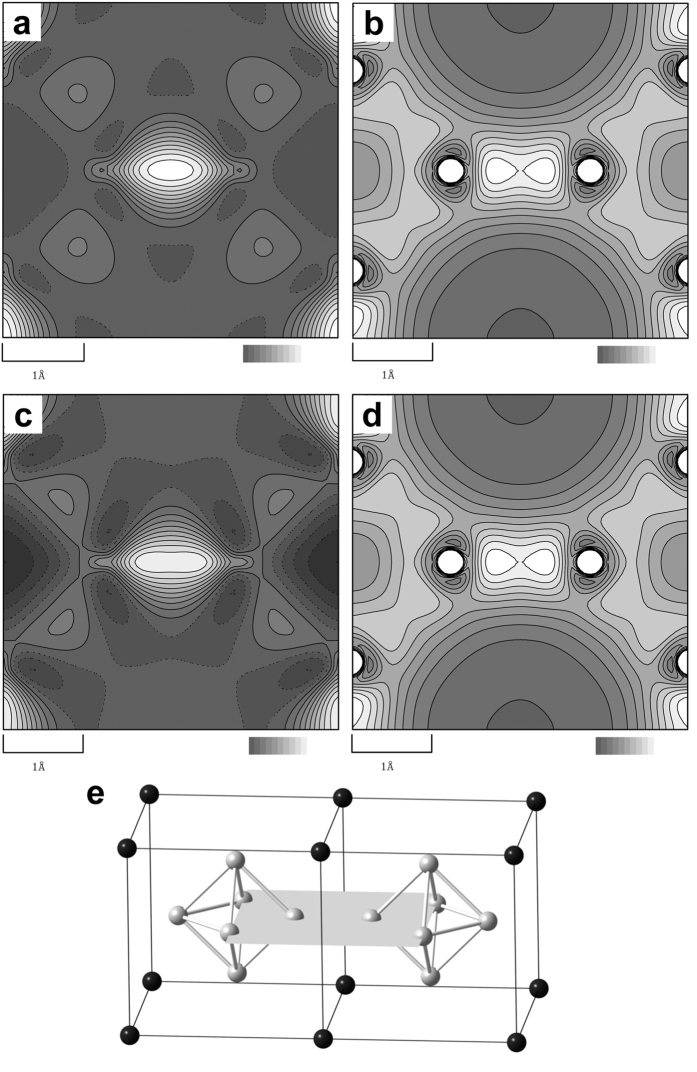
Static deformation density and valence charge density maps, schematic structure by multipole refinement and topological analysis of LaB_6_ and BaB_6_ for 020 plane. (**a**,**c**) Static deformation maps for (**a**) LaB_6_ and (**c**) BaB_6_. Contour lines are drawn from 0.0 to 0.4 with 0.04 *e/*Å^3^ step. (**b**,**d**) Valence charge density maps for (**b**) LaB_6_ and (**d**) BaB_6_. Contour lines are drawn from 0.0 to 1.0 with 0.1 *e*/Å^3^ step. Dark gray and white indicate low and high density regions respectively. Color bars are also shown at the bottom of the figures. (e), Schematic figure indicating 020 plane are also shown. The maps are drawn by the program using pgplot library (http://www.astro.caltech.edu/~tjp/pgplot/) and edited with Gimp 2.8.14 (https://www.gimp.org). The schematic figures was drawn with a program Ball&Stick^37^.

Figure 2a,b,c and d show the static deformation and valence charge density from multipole refinement for LaB_6_ and BaB_6_ as contour maps of 020 sections. The charge density section is represented schematically in Fig. 2e. The six boron atoms are located on the plane. The strong B-B bonds are clearly recognized at the center of the figures. The bond is connecting to B_6_ octahedra, what we call the B_6_-B_6_ bond. The other charge density overlaps between B atoms are also recognized at the vicinities edges, what we call the B-B bond. There are strong charge density peaks at the B_6_-B_6_ midpoint in Fig. 2a and c. There are also weak peaks at the B-B midpoints. The charge densities at the B_6_-B_6_ bond midpoints of LaB_6_ are 0.43 *e*/Å^3^ and 1.06 *e*/Å^3^ in the static deformation and valence densities, respectively. The charge densities at the B_6_-B_6_ bond midpoints of BaB_6_ are 0.39 *e*/Å^3^ and 0.99 *e*/Å^3^ in the static deformation and valence densities, respectively. The charge densities at the B-B bond midpoints of LaB_6_ and BaB_6_ are 0.08 *e*/Å^3^ and 0.08 *e*/Å^3^ in the static deformation densities, respectively.

The topological properties for LaB_6_ and BaB_6_ were calculated from the total charge density by the multipole refinements using program TOPOXD^30^. The lengths of bond paths, charge densities and Laplacians at bond critical points (BCPs) are listed in Table 2. The striking difference is found in the bond path length between LaB_6_ and BaB_6_. The B_6_-B_6_, B-B and B-*M* lengths of LaB_6_ are 1.657 Å, 1.763 Å, and 3.049 Å which are 0.089 Å, 0.014 Å, and 0.086 Å shorter than those of BaB_6_. The B_6_-B_6_ and B-*M* distances mainly contribute to the 0.11 Å difference of the lattice constant between 4.149410(2) Å for LaB_6_ and 4.258990(3) Å for BaB_6_. Structure parameters are also listed in Supplementary Table S1. The observed structural changes are consistent with previous structural studies^14^,^31^.

Chemical bonding in second row elements can be simply interpreted by the Laplacians^32^. The Laplacians at the BCP of the B_6_-B_6_ bonds are −10.07 *e*/Å^5^ for LaB_6_ and −6.77 *e*/Å^5^ for BaB_6_. These are covalent bonding interactions in both the materials. The covalent interaction from the Laplacian of LaB_6_ is almost half and that of BaB_6_ is almost one-third of that of diamond, −21.3 *e*/Å^5^. The covalency in the B_6_-B_6_ of LaB_6_ is stronger than that of BaB_6_. The Laplacian at the BCP of the B-B bond of BaB_6_ indicates also the covalent bonding interaction, −1.52 *e*/Å^5^, and the covalency of BaB_6_ at this point is stronger than that of LaB_6_, −0.52 *e*/Å^5^. In other words, the boron atom in LaB_6_ has B-B dimer-like feature than that of BaB_6_. The Laplacians of the *M*-B bonds are 1.51 *e*/Å^5^ for LaB_6_ and 1.33 *e*/Å^5^ for BaB_6_. It is still difficult to interpret the bond between heavy elements^32^. In the present charge density, several parameters cannot be refined for the heavy element. We do not describe the evaluation of the *M*-B bonds in the present paper.

The atomic basins of La and B atoms for LaB_6_ and Ba and B atoms for BaB_6_ are shown in Supplementary Figures S5a and S5b. The shape of atomic basin for La atom in LaB_6_ is very similar to that for Ba atom in BaB_6_. The triangle shape of atomic basin for B atom in LaB_6_ is almost identical to that in BaB_6_. The charges in atomic basins for La and Ba atoms were 55.17 *e* and 54.86 *e*, which are corresponding to + 1.83 *e* and + 1.14 *e*, respectively. The charges of B atoms were −0.28 *e* for LaB_6_ and −0.14 *e* for BaB_6_, respectively. It is found that the B_6_ cluster in LaB_6_ has approximately 0.7 more electrons than that in BaB_6_.

**Figure 3:**
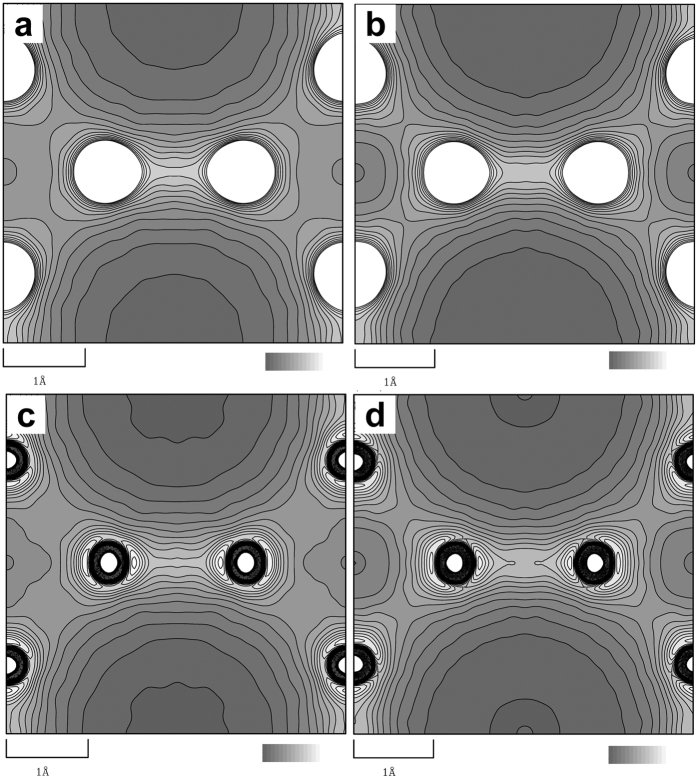
Charge densities of LaB_6_ and BaB_6_ based on the MEM. (**a**,**b**) Total charge density maps of (**a**) LaB_6_ and (**b**) BaB_6_ for 020 plane. (**c**,**d**) Valence charge densities of (**c**) LaB_6_ and (**d**) BaB_6_ by the combination of the MEM and multipole refinement. Contour lines are drawn from 0.0 to 1.5 with 0.1 *e/*Å^3^ step widths. The maps are drawn by the program using pgplot library (http://www.astro.caltech.edu/~tjp/pgplot/) and edited with Gimp 2.8.14 (https://www.gimp.org).

We successfully carried out multipole modelling of charge density connecting with topological analysis with sufficiently small reliability factors. In order to confirm the accuracy of structure factors and charge densities more precisely, we did the MEM charge density analysis of the present data. The charge densities of LaB_6_ and BaB_6_ using the observed structure factors from final powder fittings were calculated by the MEM. Figure 3a and b show the MEM charge densities of LaB_6_ and BaB_6_ for 020 plane as a contour map. The charge density at the B_6_-B_6_ bond midpoint is 1.06 *e*/Å^3^ for LaB_6_ and 1.03 *e*/Å^3^ for BaB_6_ which are much higher than those at B-B bond midpoint, 0.69 *e*/Å^3^ for LaB_6_ and 0.69 *e*/Å^3^ for BaB_6_.

In order to perform quantitative comparison between the MEM charge densities and theoretical valence density, we developed the method for determination of an experimental valence charge density by the combination of MEM and multipole refinement. The experimental valence charge densities were calculated by the subtraction of core charge densities from the total MEM charge densities. The core charge densities were calculated by MEM using *F*_core_(*h k l*) which were the structure factors of core with thermal motion calculated by the XD program and the uncertainties of observed structure factors, σ(*h k l*). Figure 3c and d show the valence charge densities for LaB_6_ and BaB_6_. The charge densities at the B_6_-B_6_ and B-B bond midpoints were 1.06 *e*/Å^3^ and 0.69 *e*/Å^3^ for LaB_6_ and 1.03 *e*/Å^3^ and 0.69 *e*/Å^3^ for BaB_6_. The charge densities at the midpoint of B_6_-B_6_ bond are much larger than that at B-B bond in both the materials. These features are consistent with the valence densities by multipole refinement and theoretical calculation. These results confirm the quality of structure factors in the present study is enough for accurate charge density studies.

**Figure 4:**
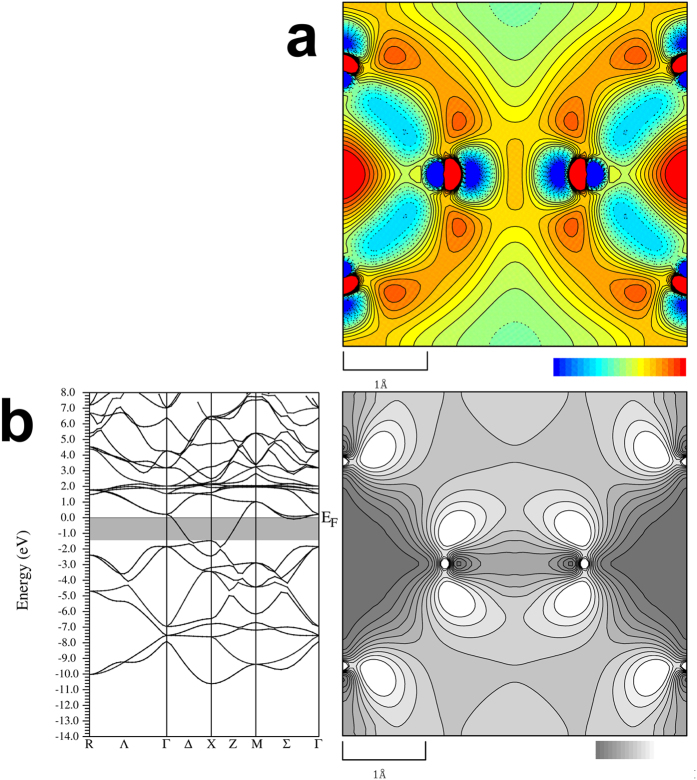
Contour map of difference of valence charge densities between LaB_6_ and BaB_6_ and Band structure plot with contour map of the theoretical electron density with energy from −1.34 to 0.0 eV. (**a**) Difference of valence charge density between LaB_6_ and BaB_6_ based on multipole refinement. Contour lines are drawn from −0.1 to 0.1 with 0.01 *e/*Å^3^ step widths. (**b**), Spin-up charge density with the band structure. The gray colored part in the band structure shows the energy of charge densities. Contour lines are drawn from 0.0 to 0.003 with 0.0003 *e/*Å^3^ step widths. The map is drawn by the program using pgplot library (http://www.astro.caltech.edu/~tjp/pgplot/) and edited with Gimp 2.8.14 (https://www.gimp.org).

The valence, deformation, and total charge densities of LaB_6_ are very similar to those of BaB_6_. The main features are the strong B_6_-B_6_ bond, B-B bond and weak *M*-B bond in both the materials. To detect small difference between LaB_6_ and BaB_6_, we calculated a difference of valence charge densities between LaB_6_ and BaB_6_. The unit cells of both the materials were divided by 85 × 85 × 85 pixels for charge density subtraction. The size of both the densities was normalized by the unit cell. Figure 4 shows the difference density as a contour map for 020 section obtained from the multipole valence densities. There are weak charge density peaks around B_6_-B_6_ bond whereas there are weak charge densities on the B_6_-B_6_ line. These facts indicate the charges along B_6_-B_6_ line are almost identical between LaB_6_ and BaB_6._ It is also clearly recognized that there are excess electrons in the center of B_6_ octahedra.

## Discussion

We have successfully detected the small difference of valence charges between LaB_6_ and BaB_6_. Two kinds of electron localized parts were found in the metallic LaB_6_. We calculated theoretical charge density of LaB_6_ using computer program WIEN2k^33^ to investigate the observed charges. A theoretical band structure of LaB_6_ calculated by the present study is shown in Fig. 4b. This is consistent with the many previous studies^9^,^10^,^11^,^12^. Then we calculated and visualized charge densities by changing the energy level from −1.36 to 0 eV, from −2.72 to −1.36 eV, and from −4.08 to **−**2.72 eV (see Supplementary Figures S6). The contour map with the energy level from −1.36 to 0 eV is also shown in Fig. 4b. The charge density localization along with B_6_-B_6_ bond is recognized in Fig. 4b. We found the weak peaks along with B_6_-B_6_ line are consistent with the electron distributions just below the Fermi level in the theoretical charge density. The band structure of BaB_6_ has been reported by several researchers^13^,^14^. The energy bands from −2.72 to −1.36 eV and −4.08 to −2.72 eV of LaB_6_ also similarly exist in the band structure below Fermi level of BaB_6_. The band from −1.36 to 0.0 eV of LaB_6_ does not exist in the reported band structure of BaB_6_. These facts confirm that the present study visualized the spatial distribution of electrons just below the Fermi level experimentally.

The charge localization in the center of B_6_ has never been found in the theoretical charge densities of Fig. 4b, S6a and S6b. The volume of B_6_ octahedron for LaB_6_, 2.582 Å^3^, is 2.5% smaller than that of BaB_6_, 2.647 Å^3^, from the present determined structure. Both the materials have bonding octahedron orbitals, such as *a*_1g_^34^. The electron density of these orbitals for LaB_6_ inside the octahedron should be higher than that of BaB_6_ due to the smaller volume. We calculated amount of the electrons observed at the center of B_6_ octahedron. The number of pixels in positive charge region inside B_6_ octahedron is 8000 which is corresponding to (*a*/3*√2)^3^. The electron density at the center of the octahedron is 0.13 *e*Å^−3^. The total number of the electron inside the octahedron is 0.06*e* which is 3% of two electron filled *a*_1g_ orbital. Therefore, the charge density in the B_6_ octahedron is mainly due to the volume difference.

In this paper, we have successfully observed the three-dimensional charge density distribution directly on and just beneath Fermi level by the X-ray charge density study. Quantitatively comparison between iso-structural solids with different electrical properties plays a crucial role for the detection. Most of the materials with exotic physical properties such as superconductor have the iso-structural and different property solids. The present experimental and analytical technique can be used for such system. Electron density distribution is now one of the most information-rich observable owing to the great improvement of experimental situation such as synchrotron X-ray source.

## Methods

### Powder sample preparation

The powder samples of 3 N purity LaB_6_ with less than 350 mesh particle size and 2N5 purity BaB_6_ with less than 100 mesh particle size were purchased from *Mitsuwa Chemicals Co*., *Ltd*. The samples were ground using an Al_2_O_3_ mortar. The fine particles with less than 5 μm size were selected by the precipitation method with ethanol as a solvent. The selected fine particles were agglomerated together with tiny amounts of glue. The samples were cut into a rectangle. By using these samples, we did not need to use glass capillary in the experiment. This is effective to reduce background scattering in diffraction data. The photographs of samples are shown in the insets of Fig. 1a and b. The size of the samples were 3.5 mm × 0.25 mm × 0.27 mm for LaB_6_ and 3.5 mm × 0.23 mm × 0.29 mm for BaB_6_ as shown in the Figures.

### Synchrotron X-ray powder diffraction measurement

The powder diffraction data were measured by a Large Debye-Scherrer camera with an Imaging Plate (IP) as a detector at BL02B2 beamline^16^. Wavelength of incident X-ray was 0.35691(3) Å calibrated using powder diffraction data of NIST CeO_2_ standard sample. The high-energy X-ray was used for reducing an effect of absorption. In the case of LaB_6_, the angular dependence of absorption between 0° and 120° at 2*θ* is less than 0.9%. The 120 ° at 2*θ* is the maximum diffraction angle in the present study. In the case of BaB_6_, the dependence is less than 0.7%. The collimator size was 3.0 × 0.5 mm. We measured two datasets using an overlaid measurement technique^24^ for LaB_6_ and BaB_6_. One of the data was measured by moving IP cassette to measure high order data, D2. The D2 data were measured by moving IP with long exposure time, 80 minutes for LaB_6_ and 120 minutes for BaB_6_. We recognized Bragg peaks at better than *d* > 0.22 Å resolution range in the D2 data. Another data, D1, using a normal procedure which includes the high intense low-angle Bragg reflections were measured with 20 minutes and 30 minutes exposure times for LaB_6_ and BaB_6_, respectively. Temperatures of samples for all the measurement were controlled at 100 K using N_2_ gas flow devices.

### Data analysis using relativistic atomic scattering factors

An analysis of ultra-high resolution data requires an external treatment. It is impossible to analyze the data by using atomic scattering factors expressed by four terms Gaussian which are listed in International Table B and used in normal crystallography software. Since the equation cannot represent high order data with *d* < 0.25 Å, we used relativistic atomic scattering factors calculated by Su and Coppens^35^. By using the scattering factors, powder fittings in high order region were drastically improved. The present compounds include relatively heavier elements. The fittings in high angle regions in the present study represent requirements and abilities of relativistic scattering factors from experiment.

The 2*θ* ranges of Rietveld analysis for LaB_6_ were from 1.9° to 67.5° for D1 and from 54.8° to 118.35° for D2. The ranges for BaB_6_ were from 1.9° to 67.37° for D1 and from 52.5° to 105.055° for D2. Reliability factors based on weighted profile, *R*_wp_, and Bragg intensity, *R*_I_, of LaB_6_ were 6.50% and 1.13% for D1, 0.75% and 0.87% for D2, and 2.27% and 0.97% for two datasets and those of BaB_6_ were 5.97% and 1.09% for D1, 0.88% and 1.28% for D2, and 2.22% and 1.22% for two datasets.

### Multipole refinement

The [1 s]^2^ were core and [2s2p]^3^ were valence electrons for boron atom for the refinement. The [Xe]^54^ were core and [6 s5d]^3^ were valence for La atom. The [Xe]^54^ were core and [6s]^2^ were valence for Ba atom. Local coordinates for B and *M* ( = La, Ba) atoms were defined for the multipole refinement. The *x*-, *y*-, and *z*- axes of the local coordinate for metal atoms were parallel to [100], [010], and [001] directions. The *x*-, *y*-, and *z*- axes for B with 4 *mm* site symmetry were [*x*, 1/2, 1], [*x*, 1, 1/2] and [−1, 1/2, 1/2] directions. The multipole populations which can be refined for *M* site are *P*_40_ and *P*_44+_ where the *P*_44+_ is 0.74045*P*_40_ from the symmetry restriction. The populations which can be refined for B site are *P*_10_, *P*_20_, *P*_30_, *P*_40_, and *P*_44+_.

### Charge density study by Maximum Entropy Method

The unit cells of LaB_6_ and BaB_6_ were divided by 128 × 128 × 128 pixels. The MEM analyses were carried out using computer program ENIGMA^36^. Total numbers of Bragg reflections were 898 for LaB_6_ and 775 for BaB_6_. Reliability factors of MEM analysis were *R* = 0.47% and *R*_W_ = 0.53% for LaB_6_ and *R* = 0.58% and *R*_W_ = 0.47% for BaB_6_.

### Theoretical calculation by WIEN2k

Theoretical band structure and charge density of LaB_6_ were calculated using full potential-linearized augmented plane wave (FP-LAPW) method with the generalized gradient approximation (GGA) by WIEN2k package^33^. The calculation was performed using structure information including lattice constants and atomic coordinates from the result of the present Rietveld refinement. We used 1000 k points with *R*^MT^*K*_max_ = 7.0, where *R*^MT^ is the Muffin-Tin radius and *K*_max_ is the maximum value of the wave vector, in the calculation.

## Additional Information

**How to cite this article**: Kasai, H. and Nishibori, E. Spatial distribution of electrons near the Fermi level in the metallic LaB_6_ through accurate X-ray charge density study. *Sci. Rep.* **7**, 41375; doi: 10.1038/srep41375 (2017).

**Publisher's note:** Springer Nature remains neutral with regard to jurisdictional claims in published maps and institutional affiliations.

## Supplementary Material

Supplementary Figures and Tables

## Figures and Tables

**Table 1:** Multipole refinement details.

		LaB_6_	BaB_6_
*M*	*P*_v_	0.47 (19)	1.4 (2)*
	*P*_40_	0.10 (10)	0.37 (9)
	*P*_44+_	0.07 (7)	0.27 (6)
	κ	1	0.97 (6)
	κ’	1	0.95 (4)
B	*P*_v_	3.09 (3)	3.10 (4)*
	*P*_10_	0.048 (16)	0.015 (12)
	*P*_20_	0.10 (2)	0.122 (18)
	*P*_30_	0.17 (4)	0.20 (3)
	*P*_40_	0.08 (5)	0.00 (3)
	*P*_44+_	0.09 (4)	0.04 (3)
	κ	0.99 (3)	0.990 (5)
	κ’	1.05 (2)	1.11 (3)
	C111	0.000050 (179)	
	C112 = C133	−0.000036 (87)	
	D1111	0.000061 (121)	
	D2222 = D3333	−0.000055 (98)	
	D1122 = D1133	−0.000038 (40)	
	D2233	0.000002 (66)	

**Table 2:** Results of Topological analysis.

Bond	Bond Path[Å]	LaB_6_	∇^2^ρ[eÅ^−5^]	Bond Path[Å]	BaB_6_	∇^2^ρ[eÅ^−5^]
		ρ[eÅ^−3^]			ρ[eÅ^−3^]	
B_6_-B_6_	1.657	1.12	−10.07	1.746	0.99	−6.77
B-B	1.763	0.71	−0.52	1.777	0.73	−1.52
B-M	3.049	0.14	1.51	3.135	0.12	1.33

The esds are not given in XD2006. It is estimated that the esds are the size as the last significant figure.
