# Supplementary material for: Spatial distribution of electrons near the Fermi level in the metallic LaB6 through accurate X-ray charge density study
Source: Sci Rep. 2017 Jan 25;7:41375. doi: 10.1038/srep41375 (PMC5264647; doi:10.1038/srep41375)
Supplement: Supplementary Figures and Tables [file srep41375-s1.pdf]

Supplementary Information

Spatial distribution of electrons near the Fermi level in the metallic LaB_6 through accurate X-ray charge density study

Hidetaka Kasai & Eiji Nishibori*

Division of Physics, Faculty of Pure and Applied Sciences, Center for Integrated Research in Fundamental Science and Engineering & Tsukuba Research Center for Interdisciplinary Materials Science, University of Tsukuba. 1-1-1,Tennodai, Tsukuba, Ibaraki, 305-8571, JAPAN

e-mail: nishibori.eiji.ga@u.tsukuba.ac.jp

Supplementary Figures

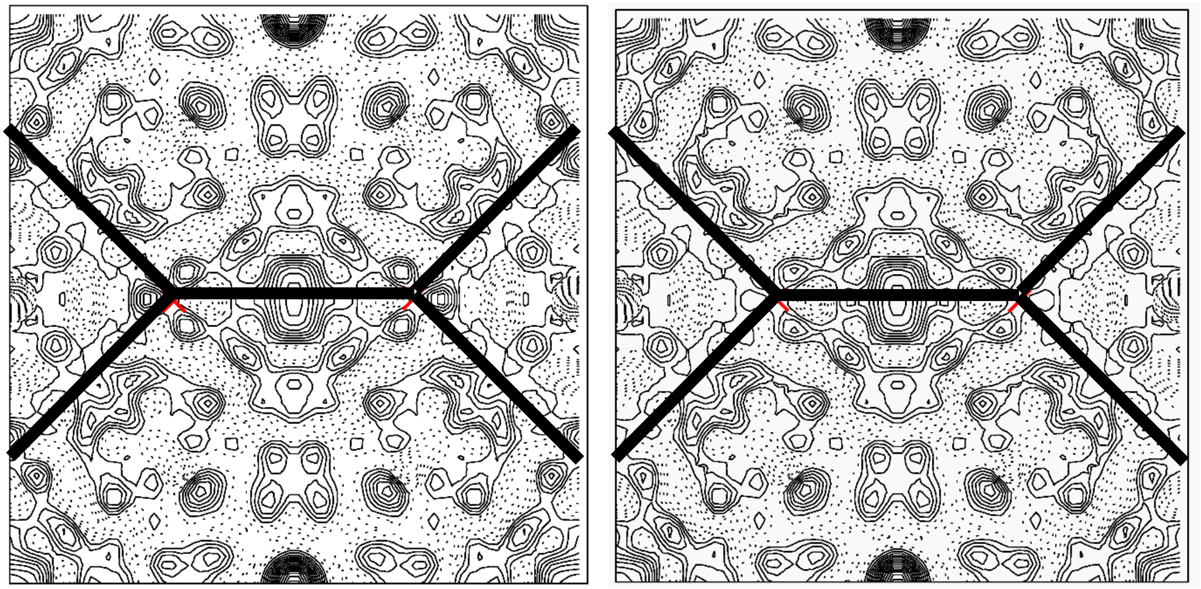

(a) (b)

Supplementary Figure S1. Sections of residual densities of LaB_6 using (a) harmonic thermal vibration model and (b) anharmonic thermal vibration model by Gram-Charlier temperature factor formalism. Contour lines are drawn from -0.75 to 0.75 with 0.05 e/Å^3 step widths. Solid lines indicate B-B bond networks.

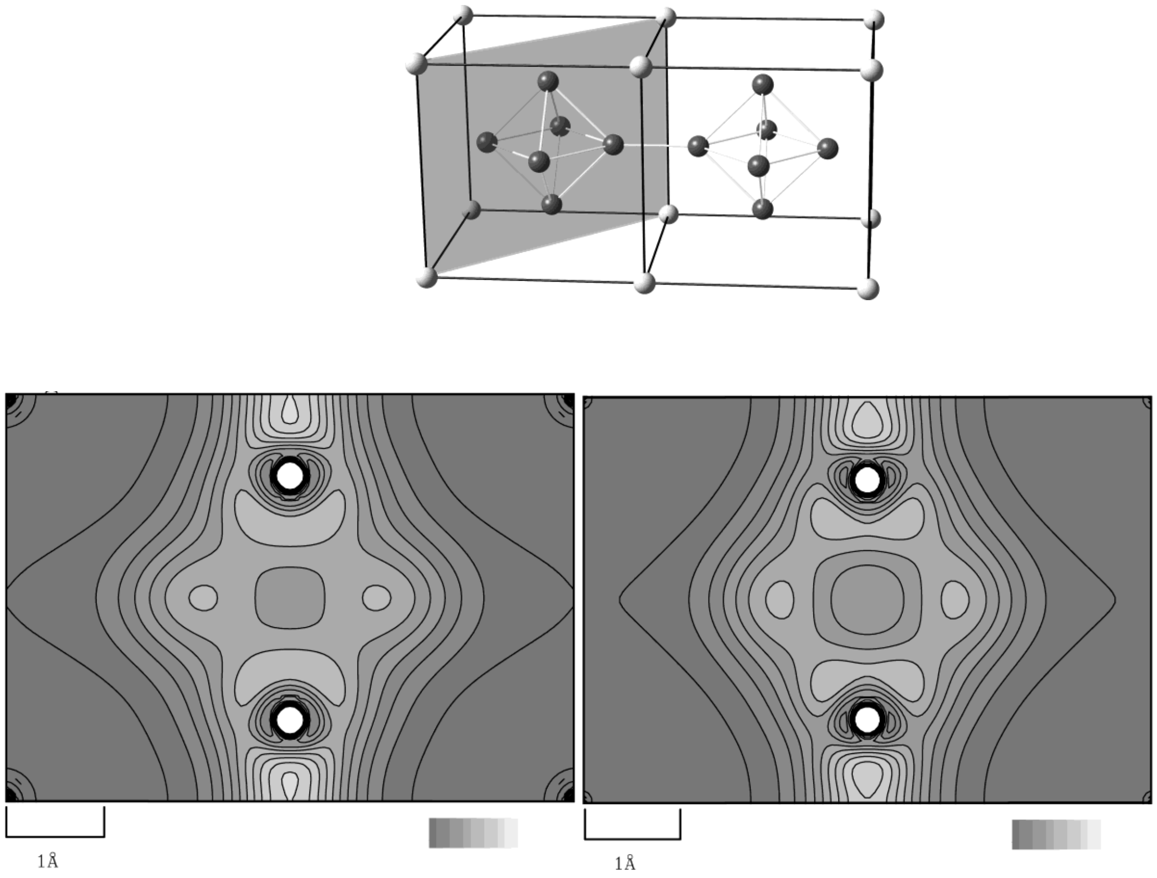

(a) (b)

Supplementary Figure S2. Sections of valence charge densities by multipole analysis. The contour maps of valence charge densities of (a) LaB_6 and (b) BaB_6 for 110 plane. There are no charge density overlaps between *M*-sites and B_6. Contour lines are drawn from 0.0 to 1.5 with 0.1 e/Å^3 step widths. The inset shows 110 plane schematically.

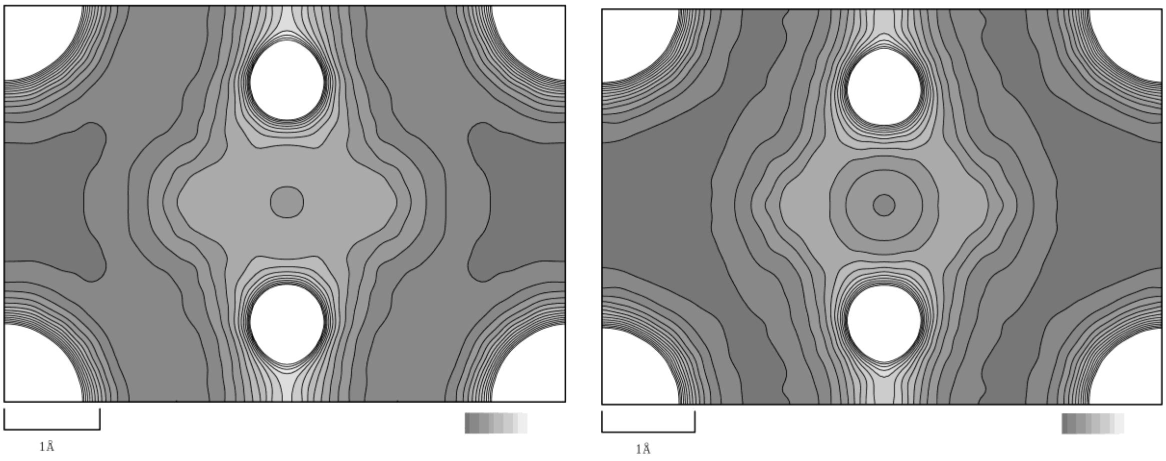

(a) (b)

Supplementary Figure S3. Sections of the MEM charge densities. The contour maps of valence charge densities of (a) LaB_6 and (b) BaB_6 for 110 plane. There are no charge density overlaps between *M*-sites and B_6. Contour lines are drawn from 0.0 to 1.5 with 0.1e/Å^3 step widths.

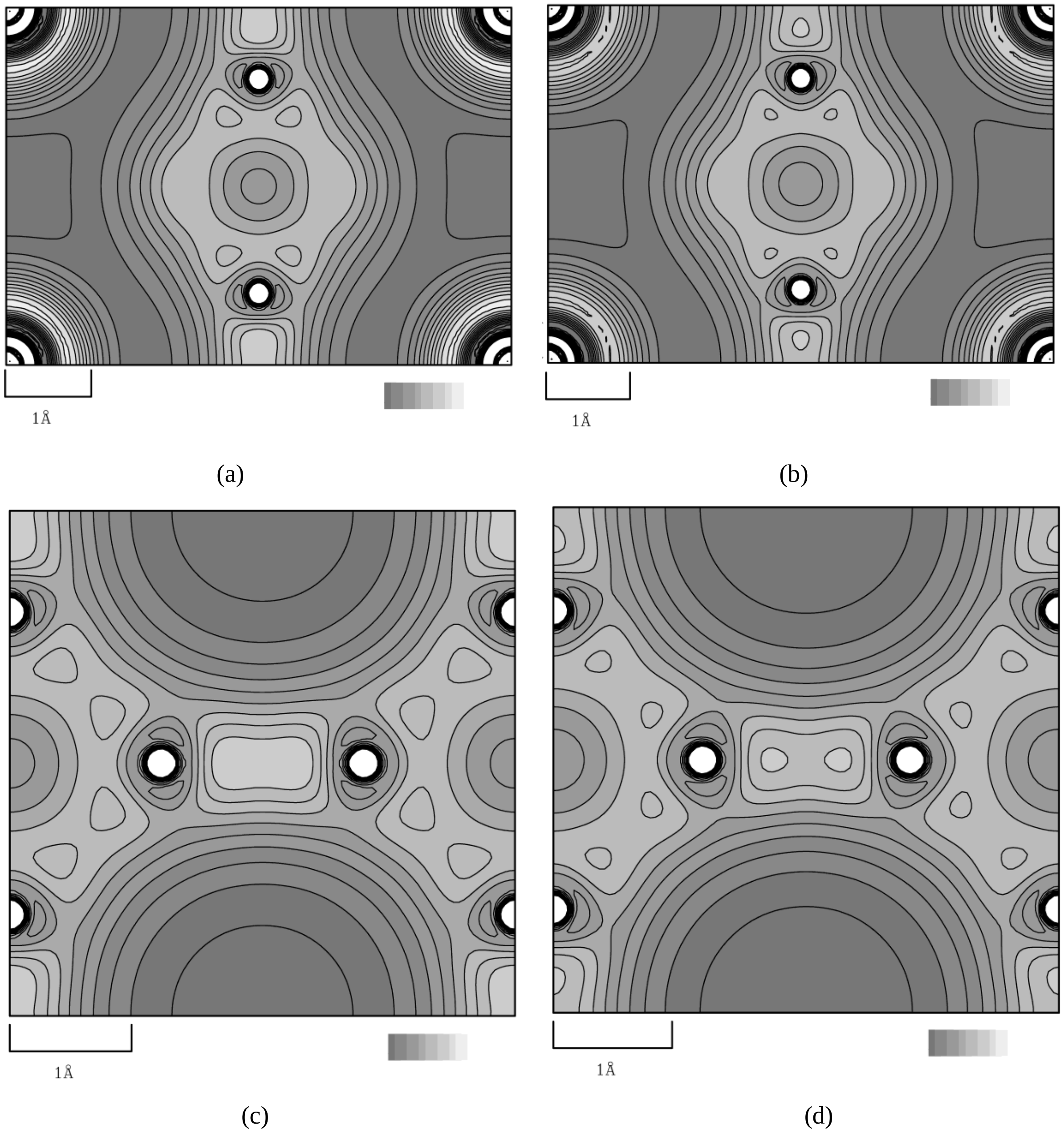

Supplementary Figure S4. Sections of the theoretical valence charge densities by WIEN2k. The contour maps of the spin-up valence charge densities of (a) LaB_6 and (b) BaB_6 for 110 plane. There are no charge density overlaps between *M*-sites and B_6. The contour maps of the spin-up valence charge densities of (c) LaB_6 and (d) BaB_6 for 020 plane. It is recognized there are two kinds bonds which are B-B and B_6-B_6. Contour lines are drawn from 0.0 to 1.5 with 0.1 e/Å^3 step widths.

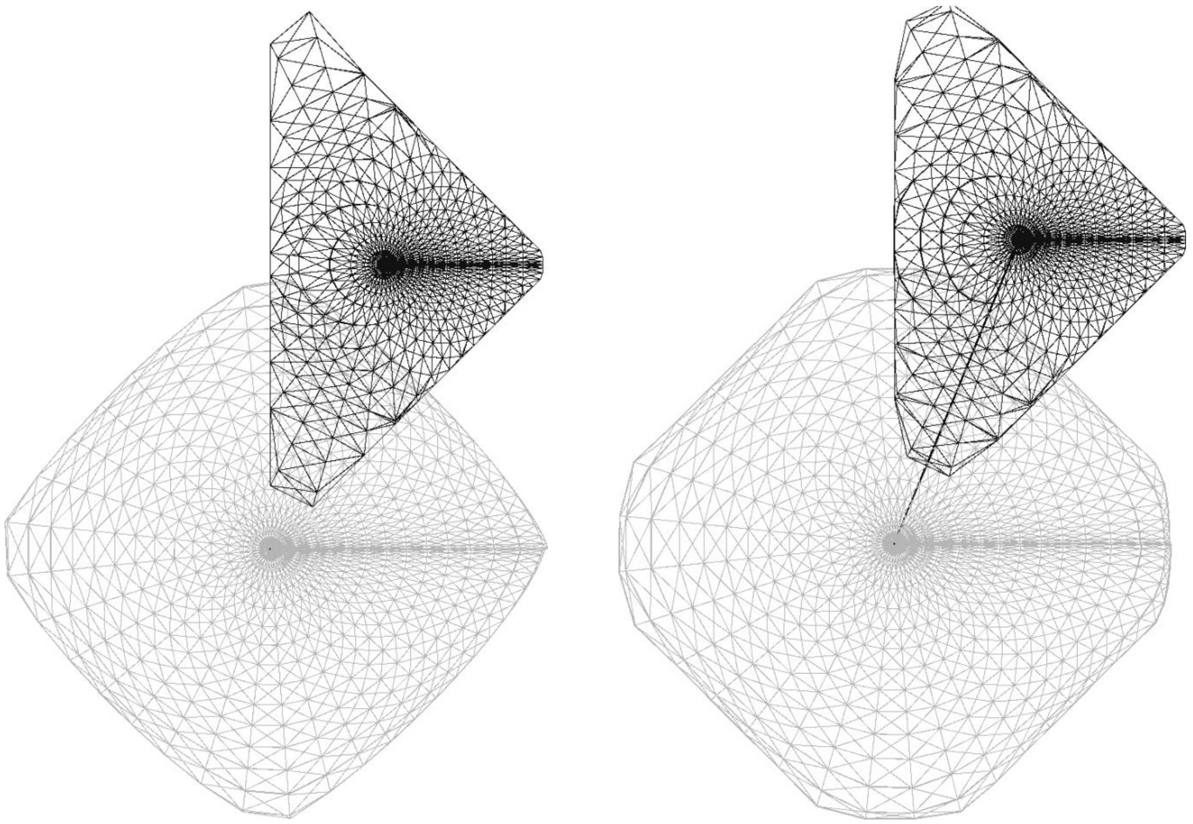

(a) (b)

Supplementary Figure S5 Atomic basins by topological analysis of charge density. The atomic basins for (a) LaB_6 and (b) BaB_6 are shown. The basins of boron atoms are triangle shape in both the materials. The basins of metal atoms are square like shape in both the materials.

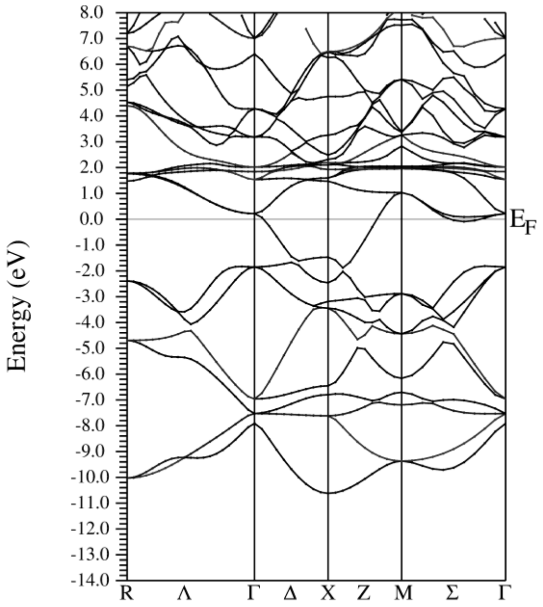

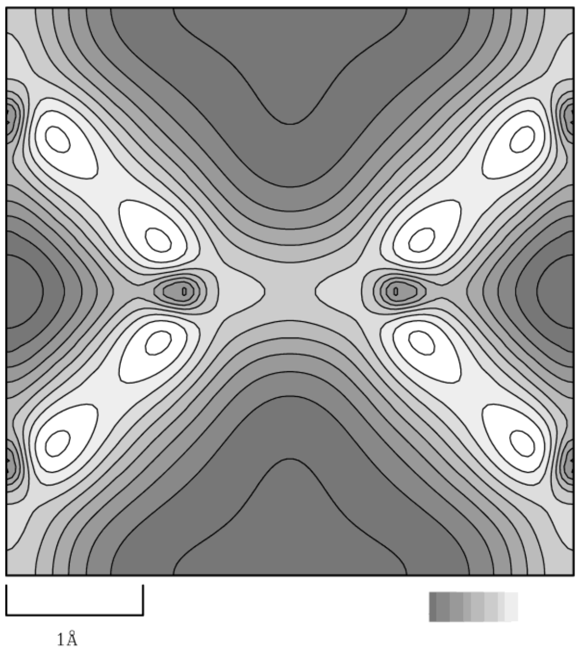

(a)

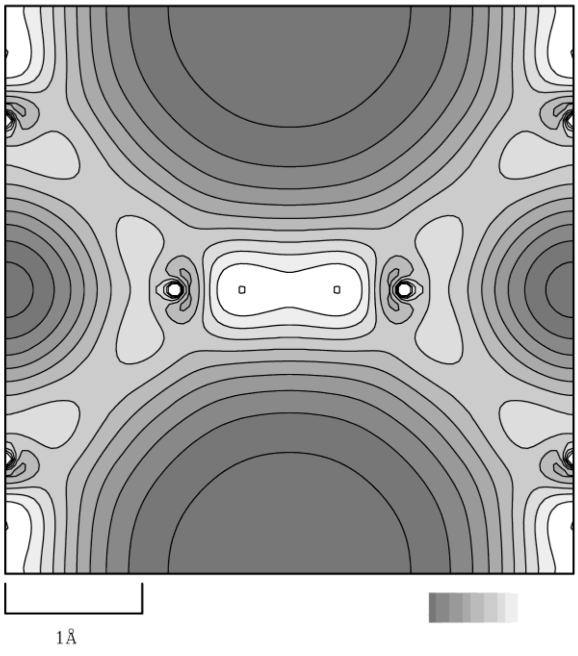

(b)

Supplementary Figure S6. Band structure plot with contour map of the theoretical electron density with energy from (a)-2.72 to -1.36 eV and (b)-4.08 to -2.72 eV. Spin-up charge density with the band structure. (a) Contour lines are drawn from 0.0 to 0.06 with 0.006 *e*/Å^3 step widths. (b) Contour lines are drawn from 0.0 to 0.1 with 0.03 *e*/Å^3 step widths.

Supplementary Table

Supplementary Table S1. Structure parameters for LaB_6 and BaB_6.

		LaB_6	BaB_6
Space Group		$Pm\bar{3}m$	$Pm\bar{3}m$
Lattice Constant		4.149410(2)	4.258990(3)
La	$U_{11}=U_{22}=U_{33}$	0.001923(19)	0.001833(2)
B	x	0.19971(9)	0.20458(9)
	U_{11}	0.00211(10)	0.00253(13)
	$U_{22}=\mathrm{U}_{33}$	0.00319(7)	0.00368(8)
